# Drugs Used in the Treatment of Viral Infections for the Prevention of Airway Remodeling in Asthma

**DOI:** 10.1155/mi/5526526

**Published:** 2025-07-24

**Authors:** Joanna Wieczfinska, Rafal Pawliczak

**Affiliations:** Department of Immunopathology, Medical Faculty, Medical University of Lodz, Lodz, Poland

**Keywords:** airway remodeling, anti-inflammatory drugs, inflammation, viral infection

## Abstract

One of the main causes of the exacerbation of chronic airway inflammatory diseases is respiratory virus infections. The most prevalent viruses that can infect humans multiple times a year are rhinovirus (RV) and respiratory syncytial virus (RSV). Because remodeling factors like matrix metalloproteinases (MMPs), which are released by infiltrating neutrophils, are present. Airway remodeling is a characteristic of the pathology of airway diseases such as bronchial asthma. In these circumstances, viral infections may result in increased neutrophilic activation, which would exacerbate asthma symptoms and modify the airway. Although a connection between viral infections and acute exacerbations of chronic inflammatory respiratory diseases has been established, anti-inflammatory medications are frequently used in conjunction with antiviral medications to treat viral infections. Although their modes of action differ, they all lessen inflammation, which is essential for the development of airway remodeling. This review addresses the potential role of anti-inflammatory and antiviral drugs in preventing airway remodeling.

## 1. Introduction—Viral Infection-Induced Respiratory Diseases

Viral infection-induced respiratory diseases are a significant cause of morbidity and mortality worldwide. These diseases affect people of all ages, but they are particularly severe in young children, elderly individuals, and people with weakened immune systems. The respiratory tract is the primary site of infection for these viruses, and they can cause a wide range of symptoms, from mild to severe. The severity of infections may also vary depending on the type of virus and the presence of other underlying health conditions [1].

Respiratory viral infections can be caused by a variety of pathogens, including RNA and DNA viruses. The most common viruses include various strains of coronaviruses (including SARS-CoV-2, responsible for COVID-19), respiratory syncytial virus (RSV), influenza viruses, and rhinoviruses (RVs) [1, 2]. These pathogens attack the epithelium of the respiratory system, leading to inflammation and tissue damage. The clinical manifestations of respiratory diseases caused by viral infections can vary, but most commonly include fever, cough, shortness of breath, sore throat, runny nose, headache, and fatigue. For more serious infections, complications such as pneumonia, respiratory failure, and others may occur [3].

This study aims to review the potential of drugs used in the treatment of viral infections to serve as therapeutic interventions targeting airway remodeling processes induced by viral infections.

## 2. Viral Infections in the Development of Airway Remodeling in Asthma

Airway remodeling in asthma, defined as structural changes in the airway wall, is a key feature of asthma and is thought to contribute to the development of irreversible airflow limitation. Airway remodeling is characterized by various structural changes in the airway wall, including subepithelial fibrosis, increased smooth muscle mass, goblet cell hyperplasia, and mucus hypersecretion [4]. Chronic airway inflammation and airway injury are assumed to be the primary causes of these structural alterations, as they activate different cell types such as smooth muscle, fibroblast, and epithelial cells. Numerous mediators, such as growth factors, cytokines, and extracellular matrix (ECM) proteins, are released by activated cells to aid in tissue remodeling and repair [5, 6]. Asthmatic airways are characterized by increased deposition of ECM proteins in the submucosa, lamina propria, and reticular basement membrane region. This deposition thickens the airway wall and obstructs airflow. The most prevalent components of the ECM in the asthmatic lung are collagen fibers, fibronectin, and tenascin [7]. When ECM proteins accumulate abnormally, tissue shape and function are altered, which plays a part in asthmatic airway remodeling. The primary source of ECM is fibroblasts. Asthmatic airways activate fibroblasts, which generate copious amounts of ECM [8]. Additionally, research has demonstrated that naïve lung fibroblasts are stimulated by asthmatic airway epithelial cells to make collagen, fibronectin, and transforming growth factor beta (TGF-β), a profibrotic mediator [9]. In asthma, smooth muscle cells in the airways show specific molecular changes, such as lower levels of cAMP and higher levels of mitochondrial activity and Erk1/2 MAPK expression. These changes may influence the activity of mesenchymal cells in the airway wall [10]. Immediate airflow limitation caused by remodeling of the airways has been linked to poor symptom control and no response to treatment. According to a theoretical model of airway remodeling, inflammation alone is enough to cause the airway wall to thicken toward the lumen. However, when both inflammation and contractile forces in the airways are modeled together, the thickening of the airway walls is greater, suggesting that these mechanisms interact with each other [11]. Furthermore, a number of mice models have indicated a possible connection between remodeling and inflammation, which will be discussed further below [12–14].

There is continuous discussion and investigation regarding the role that viral infections play in the development of airway remodeling in asthma. The structural alterations to an asthmatics' airways, including thickening of the airway wall, increased smooth muscle mass, mucus hypersecretion, and fibrosis, are referred to as airway remodeling. These alterations may worsen asthma symptoms and intensity, impair lung function, and raise the possibility of exacerbations [15, 16].

One of the most frequent causes of asthma exacerbations, particularly in children, is viral infections. Asthma development and progression are linked to viral respiratory infections primarily caused by RV and RSV [17]. These viruses can damage the airway epithelium and subepithelial tissues by causing inflammation, oxidative stress, and immunological responses in the upper or lower respiratory tract. The most common cause of viral respiratory infections and the virus most often linked to airway remodeling is RV [18]. Inflammatory pathways that are activated by RV infections are linked to the pathophysiology of airway inflammation and the exacerbation of long-term respiratory conditions like asthma and chronic obstructive pulmonary disease (COPD) [19]. RV infection causes immune cells and airway epithelial cells to produce inflammatory mediators like chemokines, cytokines, and mucin. These mediators can cause bronchial hyperresponsiveness, mucus secretion, airway obstruction, and secondary bacterial infection [20–22]. Cytokines produced by RV infection, including IL-5, IL-13, IL-25, and IL-33, have been linked to severe and long-lasting asthma [23]. Medications for inflammation caused by RV infection mainly focus on relieving symptoms and preventing complications, rather than curing or stopping the infection itself [24].

RV infections are usually harmless and resolve after a few days; nevertheless, in the context of respiratory remodeling, they can have serious consequences. Some studies have suggested that viral infections in early life can induce or enhance airway remodeling by stimulating the secretion of growth factors that promote cell proliferation, ECM deposition, and angiogenesis in the airways [16, 25, 26]. RV infection in young children increased the levels of TGF-β1, vascular endothelial growth factor (VEGF), and platelet-derived growth factor (PDGF) in nasal secretions [26, 27]. These growth factors are known to be involved in airway remodeling and asthma pathogenesis. RSV infection activates innate immune receptors such as toll-like receptors (TLRs) and retinoic acid-inducible gene I (RIG-I) in airway epithelial cells and immune cells, which leads to the production of inflammatory mediators such as cytokines, chemokines, reactive oxygen species, and mucin [28–31]. These mediators can cause airway obstruction, mucus secretion, bronchial hyperresponsiveness, cell death, and secondary bacterial infection [32]. In newborns with upper and lower respiratory tract infections, RSV induces a neutrophil-intensive inflammation of the airway. Eosinophilia is a possible side effect of infection that is more prominent in the most severe RSV LRTI (lower respiratory tract infection) cases [33, 34]. Like many other common respiratory viruses, the severity of the disease is correlated with the RSV viral load [35].

The mechanisms by which viral infections contribute to the development of asthma are complex and involve a number of immunological and inflammatory processes [36]. Viruses can induce inflammatory responses in the airways by activating inflammatory cells such as eosinophils, neutrophils and Th2 lymphocytes, leading to the production of pro-inflammatory cytokines such as interleukins (IL-4, IL-5, IL-13) and interferon gamma (IFN-γ) [5, 15, 36, 37]. These cytokines can promote bronchial hyperresponsiveness, overproduction of mucus, and increased smooth muscle contraction in the airways, leading to asthma symptoms. Numerous studies have demonstrated that HRV infection increases expression of the primary epithelial mucin MUC5AC [38–40]. Increased mucin production and mucus hypersecretion are also common features of airway remodeling in asthma. Moreover, elevated release of MUC5AC from epithelial cells has been observed in vivo during experimental HRV infections. Activation of the transcription factor, ERK mitogen-activated protein kinase, and epidermal growth factor receptor have all been connected to the induction of MUC5AC in HRV-infected epithelial cells [39, 40].

The remodeling process, which begins with infection of the airway epithelium, recognizes the virus through pattern recognition receptors (PRRs) such as TLR3, RIG-I and MDA5. Activation of these receptors leads to the induction of an antiviral response and the secretion of pro-inflammatory cytokines (IL-6, IL-8) and chemokines (CCL5, CXCL10) [26, 41]. This response results in the recruitment of inflammatory cells, including eosinophils, monocytes, and T lymphocytes, which enhance local inflammation. At a later stage, the structural cells of the bronchial wall—mainly fibroblasts and smooth muscle cells—are activated under the influence of inflammatory mediators and growth factors such as TGF-β1, FGF-2, VEGF, or EGF [41]. These cytokines stimulate fibroblast proliferation, their differentiation into myofibroblasts, and the production of ECM components, mainly type I and III collagen [42]. In parallel, there is increased expression of matrix metalloproteinases (MMP-9) and their inhibitors (TIMP-1), which disrupts the balance of ECM degradation and restoration, leading to thickening of the basement membrane and loss of airway elasticity [43]. Importantly, viral infection can also induce neovascularisation through increased VEGF production, another element of remodeling observed in biopsies from patients with asthma [27]. In the context of asthma, rhinoviral infection may also exacerbate airway remodeling through induction of cup cell metaplasia, increased mucus production and activation of signaling pathways associated with type 2 responses (e.g., IL-33, TSLP). These changes lead to chronic inflammation and structural modifications in the airways, which may contribute to disease progression [44]. RV infection initiates a series of inflammatory responses, starting with infection of airway epithelial cells. These cells secrete a variety of mediators, including type 2 cytokines, chemokines, and growth factors, which have paracrine effects on neighboring subepithelial cells, inducing responses that lead to tissue remodeling [18]. For instance, RV exposure has been shown to induce epithelial-to-mesenchymal transition (EMT), leading to morphological changes in epithelial composition and contributing to airway remodeling [45].

## 3. Anti-Inflammatory Effect of Drugs Used in Viral Infections

### 3.1. Antivirals

Antiviral drugs have been traditionally designed to target viral replication, but recent evidence suggests that they may also have anti-inflammatory effects [46]. Their anti-inflammatory effects may be mediated by various mechanisms, including the inhibition of pro-inflammatory cytokines, the modulation of immune cell function, and the regulation of intracellular signaling pathways. For example, IFNs, which are used to treat viral infections, can activate immune cells and induce the production of anti-inflammatory cytokines, such as IL-10, while inhibiting the production of pro-inflammatory cytokines, such as tumor necrosis factor (TNF)-α and IL-6 [47, 48]. Similarly, nucleoside analogs, such as ribavirin, have been shown to inhibit the production of pro-inflammatory cytokines by blocking the activation of transcription factors, such as nuclear factor kappa B (NF-κB) and activator protein-1 (AP-1) [49, 50].

Antiviral drugs function by selectively targeting viral replication processes while minimizing cytotoxicity to host cells. One of the primary mechanisms involves the inhibition of reverse transcription, preventing the conversion of viral RNA into DNA and thereby halting genome integration and replication [51]. Another key strategy includes the inhibition of viral proteases, which blocks the processing of polyproteins necessary for the assembly of functional viral components. Polymerase inhibition constitutes a further mechanism, wherein the drug interferes with the synthesis of viral nucleic acids, frequently resulting in premature termination of replication [52]. Antivirals may also inhibit enzymes responsible for the release of progeny virions from infected cells, thus disrupting the propagation of the infection. Additionally, some agents induce lethal mutagenesis or impair nucleotide metabolism, compromising the fidelity of viral genome replication [53]. Few antiviral treatments seemed to be promising in clinical trials—viral entry inhibitors pirodavir, pleconaril, tremacamra, and vapendavir were among the few antivirals to be tested in humans. While they all generally shortened the duration of symptoms, they either did not offer a general benefit or did not meet study objectives. Nevertheless, only vependavir was tested in asthmatic patients, and despite being the most effective antiviral in clinical trials to date [54]. Pleconaril has not been approved for use, partly due to its increased activity of the cytochrome P450 enzyme, which increases the risk of adverse drug interactions [55]. Furthermore, no RV antiviral currently on the market has been shown to reduce airway remodeling symptoms [18].

The main viruses that cause airway remodeling are RV and RSV, but antiviral drugs used for other viruses, such as influenza or SARS-CoV-2, might also help reduce inflammation.. Some antiviral drugs, such as protease inhibitors, exhibit anti-inflammatory effects by blocking the activation of transcription factors, such as NF-κB, which are crucial for the expression of pro-inflammatory genes. By inhibiting these signaling pathways, protease inhibitors can reduce inflammatory responses in response to viral infections [56, 57]. Additionally, IL-6 and IL-8 levels have been reported to decrease in this type of antiviral [58]. In contrast, some antiviral drugs, such as the neuraminidase inhibitors used to treat influenza, may exhibit anti-inflammatory effects by modulating the immune response. They can inhibit the activation of inflammatory cells, such as macrophages and T lymphocytes, and reduce the production of pro-inflammatory cytokines, leading to a reduction in the severity of inflammatory responses in tissues [59–61].

Several antiviral medications are currently approved for the treatment of influenza. These medications include oseltamivir, zanamivir, peramivir, and baloxavir marboxil. They work by inhibiting the neuraminidase enzyme of the influenza virus, which is essential for the release of new viral particles from infected cells [62]. Studies have shown that these medications can reduce the severity and duration of influenza symptoms, but their effects on inflammation are less clear. As anti-influenza drugs do not present anti-inflammatory properties, clinical treatment of influenza should be focused on both antiviral and anti-inflammatory therapies [63]. Several antiviral drugs have been studied for their potential to reduce inflammation in COVID-19 patients. Remdesivir, an antiviral drug that targets the replication of the virus, has been shown to reduce the time to recovery in hospitalized COVID-19 patients. This drug works by inhibiting the replication of the virus by blocking its RNA polymerase. However, the effect of remdesivir on inflammation, according to Gilzad-Kohan and Jamali [64] is indirect—by reducing the amount of virus in the body. Like every group of drugs, antivirals may cause a range of adverse effects, including neuropsychiatric symptoms such as irritability, depression, and hallucinations, as well as hematologic issues like anemia and neutropenia [65]. For instance, ribavirin therapy may result in dose-dependent reversible intravascular hemolytic anemia in ~10% of patients. Additionally, high-dose ribavirin has been associated with hypomagnesemia and bradycardia during outbreaks of severe acute respiratory syndrome. Adefovir can lead to dose- and time-dependent nephrotoxicity, even at low doses. Tenofovir has been linked to gastrointestinal effects, headache, decreased bone mineral density, and nephrotoxicity [66].

### 3.2. Corticosteroids

Corticosteroids are potent anti-inflammatory agents that have been shown to improve asthma symptoms and reduce exacerbations. Though their effects on airway remodeling in asthma are still not fully understood, several studies have shown that corticosteroids can reduce airway inflammation, which may prevent or reverse some of the structural changes associated with airway remodeling [67]. Glucocorticoids are steroid hormones that exert their primary effects by binding to the intracellular glucocorticoid receptor (GR), a member of the nuclear receptor superfamily [68]. The primary mechanism of action of corticosteroids involves binding to specific intracellular receptors (GRs for glucocorticoids and mineralocorticoid receptors for mineralocorticoids) in the cytoplasm. Upon activation, the glucocorticoid–GR complex translocates to the nucleus, where it regulates gene expression by binding to glucocorticoid response elements (GREs) or by interacting with other transcription factors such as NF-*κ*B and AP-1 [69]. This results in the suppression of pro-inflammatory cytokines, adhesion molecules, and enzymes like COX-2 and iNOS, leading to strong anti-inflammatory and immunosuppressive effects. In addition to these genomic actions, glucocorticoids can also elicit rapid, nongenomic responses that are independent of nuclear GR activity [70]. These multifaceted mechanisms make glucocorticoids a cornerstone in the treatment of inflammatory and autoimmune diseases. Corticosteroids primarily function by inhibiting a number of activated inflammatory genes, which code for adhesion molecules, inflammatory enzymes, receptors, cytokines, and chemokines [71, 72]. Proinflammatory transcription factors like activator protein-1 and nuclear factor-κB (NF-κB) activate inflammatory genes in the airways by interacting with coactivator molecules like CREB-binding protein, which has intrinsic histone acetyltransferase activity. They also activate other genes related to inflammation [73]. Apart from modulation of many genes relevant to understanding their action in asthma, at a cellular level, inhaled corticosteroids (ICSs) reduce the numbers of inflammatory cells in asthmatic airways, including eosinophils, T-lymphocytes, mast cells, and dendritic cells [67]. Corticosteroids work by preventing the recruitment of inflammatory cells into the airway by suppressing the synthesis of adhesion molecules and chemotactic mediators and restricting the ability of inflammatory cells to survive in the airways [74].

The concept that using steroids in vivo would reduce virus-induced inflammation is supported by the fact that *in vitro* steroids inhibit the release of cytokines induced by RVs and RSV [75–77]. Corticosteroids have been shown to reduce subepithelial fibrosis, smooth muscle mass, and goblet cell hyperplasia in animal models of asthma. In addition, clinical studies have shown that corticosteroids can reduce airway hyperresponsiveness, which is a hallmark of asthma and is thought to be a consequence of airway remodeling [78]. Airway remodeling and inflammation are both impacted by glucocorticoid actions in ASM (i.e., ASM immunomodulatory role) [79, 80]. Additionally, glucocorticoids can control how the ASM contracts by two possible pathways—the first one is by decreasing the cholinergic hypersensitivity in the airways, probably by controlling the expression of G protein-coupled receptors (muscarinic: M2, M3; histamine: H1); the second possibility is through mediation of airway contraction or increasing airway relaxation by upregulating the *β*2AR (*β*2 adrenergic receptor) and modulating the activity of adenylyl cyclase [81].

As a first-line treatment for preventing inflammation linked to asthma, corticosteroids are well-established. Without clinical interventions, severe exacerbations of asthma can be fatal. In particular, high-dose ICS can lower exacerbation events in patients with severe disease by about 20%–25% [82]. In the previous work, Olivieri et al. reported that short-term treatment with low-dose fluticasone propionate reduced inflammatory cell infiltration into the *lamina propria* in bronchial biopsy specimens. Moreover, short-term low-dose fluticasone propionate treatment may control the intensity of airway remodeling in mild asthma [83]. In the context of RV infection, corticosteroids have been shown to reduce the severity and duration of symptoms, including cough, wheezing, and shortness of breath [84]. Fluticasone proprionate reduced RV-induced goblet cell hyperplasia and mucin production *in vitro*, and RV-induced VEGF in BEAS-2B cells [85, 86]. Budesonide suppressed RV-induced CXCL8, IP-10, and VEGF production and reduced MMP-8 production *in vitro* and in moderate asthma, respectively [77, 87].

The use of corticosteroids to treat COVID-19 has also become a popular option. Beigel et al. [88] suggest a potential additive effect when dexamethasone and remdesivir are used in the same therapeutic regimen. Corticosteroids have been extensively studied for their role in reducing inflammation in COVID-19 patients. Dexamethasone has been shown to reduce the risk of death in COVID-19 patients who require *supplemental* oxygen or mechanical ventilation [89]. It must be noted however, that corticosteroid therapy is associated with a range of adverse effects, including osteoporosis, avascular necrosis, cardiovascular events, gastrointestinal bleeding, diabetes mellitus, psychiatric disorders, and increased susceptibility to infections. The risk of these complications increases with higher cumulative doses and prolonged use [90].

### 3.3. Interferons

IFNs are cytokines primarily secreted by virus-infected cells and play a pivotal role in initiating the antiviral immune response. Upon binding of type I IFNs (e.g., IFN-α, IFN-β) to the IFNAR receptor, the JAK-STAT signaling pathway is activated, leading to the transcription of numerous interferon-stimulated genes (ISGs), including the activation of protein kinase R (PKR). PKR is a double-stranded RNA-dependent kinase that inhibits viral replication by phosphorylating the eukaryotic initiation factor eIF2*α*, thereby blocking translation. Type II IFN (mainly IFN-γ) enhances antigen presentation through MHC molecules and activates macrophages, thus supporting both innate and adaptive immunity [91]. Through these mechanisms, IFNs orchestrate a comprehensive antiviral response and contribute to pathogen elimination [92]. In addition to their antiviral properties, IFNs exhibit nofig anti-inflammatory effects in various cell types. For instance, IFN-β has been shown in microglial cells and astrocytes to suppress the production of pro-inflammatory cytokines such as TNF-α and IL-1β via inhibition of the NF-κB signaling pathway [93]. In human monocytes, IFN-α has been observed to upregulate the expression of IL-10, an anti-inflammatory cytokine, contributing to the resolution of inflammation during chronic viral infections [94]. Moreover, transcriptomic analyses of epithelial cells treated with type I IFNs revealed downregulation of inflammasome-related genes, including NLRP3, suggesting an additional regulatory mechanism in tissue-specific contexts [95]. These findings underscore the dual role of IFNs—not only as antiviral agents but also as modulators of immune homeostasis. Previous studies reported that IFN-*γ* prevents human monocytes stimulated with IL-2, IL-1, or LPS from expressing IL-8 [96, 97]. Furthermore, in human polymorphonuclear leukocytes, IFN-γ inhibits the production of IL-8 induced by LPS and TNFα [98].

Moreover, literature indicates that the interaction between IFNs and IL-1 is essential for preserving the sensitive equilibrium of the innate inflammatory response. Research has demonstrated that IFN-α and IFN-β can both suppress the transcription of IL-1α and IL-1β and stop the inflammasome from processing and producing bioactive IL-1 [99, 100]. Depending on the circumstances, the IFN-mediated control of IL-1 activity during pathogen infections can have conflicting effects. A strong immune response by the host against the pathogen may occasionally be hampered by IFNs suppression of IL-1 activity. On the other hand, IFN-mediated IL-1 suppression can be helpful in reducing tissue damage and inflammation in circumstances where high IL-1 activity could result in immunopathology [100, 101]. IFN therapy, particularly with IFN-alpha (IFN-α), is associated with a range of adverse effects, notably neuropsychiatric disturbances such as depression, anxiety, and cognitive impairment. These effects can manifest early or develop over time, potentially leading to dose reduction or discontinuation of treatment. The incidence and severity of these side effects are influenced by factors like dosage, treatment duration, and individual patient susceptibility [102].

### 3.4. NSAIDs

Nonsteroidal anti-inflammatory drugs (NSAIDs) exert their pharmacological effects primarily through the inhibition of cyclooxygenase (COX) enzymes, which are responsible for converting arachidonic acid into prostaglandins (PGs) and thromboxanes (TXs), key mediators of inflammation, pain, and fever [103]. COX-1 is constitutively expressed and involved in maintaining physiological functions such as gastric mucosal integrity and platelet aggregation, while COX-2 is inducible and primarily associated with inflammatory responses. The inhibition of COX enzymes by NSAIDs leads to a decrease in the synthesis of pro-inflammatory PGs, notably PGE_2_ and PGI_2_, which are involved in vasodilation, increased vascular permeability, and the recruitment of inflammatory cells to sites of injury [104]. By reducing the levels of these mediators, NSAIDs effectively alleviate symptoms associated with inflammation, such as pain and swelling. However, the inhibition of COX-1 by NSAIDs can result in adverse effects, including gastrointestinal irritation and bleeding, due to the reduction in protective PGs in the gastric mucosa [103, 105]. To mitigate these risks, selective COX-2 inhibitors have been developed, aiming to provide anti-inflammatory benefits while sparing COX-1-mediated protective effects.

In addition to their primary anti-inflammatory effects, NSAIDs have been shown to influence various physiological processes through their action on COX enzymes [106]. Selective COX-2 inhibitors, such as meloxicam, have demonstrated a higher affinity for COX-2 over COX-1, potentially reducing gastrointestinal side effects associated with traditional NSAIDs [107]. However, the long-term use of COX-2 selective inhibitors has raised concerns due to potential cardiovascular risks. Additionally, studies have indicated that the inhibition of both COX-1 and COX-2 can lead to a significant decrease in prostaglandin E_2_ (PGE_2_) levels, which may exacerbate conditions like inflammatory bowel disease [107, 108].

NSAIDs family includes some drugs that are suitable for treating viral respiratory infections because they have both anti-inflammatory and antiviral properties [109, 110]. Even though these drugs have been used for years to treat respiratory infections, it is still unclear which of the compounds is the safest and most effective. By suppressing the expression of IL-4 in T cells (CD4) and preventing nonimmune cells from producing IFN-*γ* at first, COX inhibitors can influence the adaptive immune response, which is a useful tactic against viral infection [111, 112].

The use of NSAIDs in the context of influenza infection has been controversial, as some studies have suggested that these drugs may exacerbate inflammation and prolong the duration of illness. However, RSV and influenza virus infections are characterized by a notable elevation in COX-2 (PTGS2) expression and COX-derived metabolites [113, 114]. RSV-induced lung pathology was reduced by pharmacologic inhibition of the COX pathway, though this was not associated with a particular metabolite [113, 115]. There has been discussion regarding the use of NSAIDs in COVID-19 patients. While some early research suggested that NSAIDs might make COVID-19 symptoms worse, other studies found no evidence of adverse effects. Ibuprofen increases the SARS-CoV-2 viral receptor, ACE2, which increases the virus ability to enter cells [116, 117]. The use of NSAIDs is associated with several potential adverse effects. These include gastrointestinal complications such as ulceration and bleeding, renal impairment leading to acute kidney injury, and cardiovascular risks including increased blood pressure and potential heart failure exacerbation. The risk of these adverse effects is heightened in individuals with pre-existing conditions, prolonged use, and dosages [118].

### 3.5. LABA and LAMA

The two main classes of bronchodilators that are currently the main medications control of asthma symptoms are long-acting beta agonists (LABA) and long-acting muscarinic antagonist (LAMA). LABA relaxes smooth muscle in the airways by binding to beta2-adrenergic receptors [119, 120]. This link modifies the postsynaptic *β*2 receptor on smooth muscle cells in the airways, activating a stimulatory GTP-binding protein (G2) that results in bronchodilation. As a result, there is an increase in cyclic adenosine monophosphate (cAMP) synthesis and adenylyl cyclase activity. The activation of protein kinase A and subsequent intracellular processes, such as the inhibition of myosin light chain kinase (MLCK), result in reduced contractility of smooth muscle in the airways [120, 121]. LAMAs, including tiotropium and umeclidinium, bind to muscarinic receptors (M3 subtype) on airway smooth muscle cells, preventing acetylcholine from binding and activating the Gq protein-coupled receptor pathway. This inhibition reduces intracellular calcium levels, leading to decreased activation of myosin light-chain kinase and subsequent bronchodilation [122]. The combination of LABA and LAMA therapies offers complementary mechanisms of action, leading to enhanced bronchodilation. Bucher et al. [123] reported that tiotropium, which blocks M3 muscarinic receptors on ASM cells, thereby preventing bronchoconstriction, has anti-inflammatory effects in mice exposed to cigarette smoke (CS) and infected with RSV or influenza virus. It significantly reduced total neutrophils and cytokines such as IL-6 in the lungs of CS-exposed mice; moreover, it may reduce the frequency and severity of exacerbations by lowering the production of IL-6 and IFN-γ in the lungs of patients.

Recent studies have demonstrated that LABAs, such as olodaterol, exert anti-inflammatory effects in airway epithelial cells by suppressing the production of pro-inflammatory cytokines like IL-8. These effects are mediated through *β*_2_-adrenergic receptors and involve the inhibition of nuclear factor-kappa B activation, a key transcription factor in inflammation. This suppression of inflammation is particularly relevant in conditions like COPD, where airway inflammation plays a central role in disease progression [124]. Similarly, LAMAs, including tiotropium and umeclidinium, have been shown to possess anti-inflammatory properties beyond their bronchodilatory effects. These agents inhibit muscarinic receptors on various cell types, including neutrophils, macrophages, and airway smooth muscle cells, leading to a reduction in the release of pro-inflammatory mediators and a decrease in airway inflammation. For instance, tiotropium has been reported to reduce cytokine and chemokine synthesis and release, as well as the number of inflammatory cells in both in vivo and in vitro models [125]. Tiotropium bromide caused a modest reduction in RV-induced goblet cell hyperplasia and mucin production in vitro, and formoterol reduced MMP9 expression in BEAS-2B cells [85, 126, 127]. In case of coronaviruses, Yamaya et al. [128] reported that COVID-1929E replication is inhibited by glycopyrronium, formoterol, and a combination of glycopyrronium, formoterol, and budesonide. These drugs also modulate infection-induced inflammation in the airway. Moreover, recent studies have shown that patients with SARS-CoV-2 COVID-19 infections who received ICS/LABA MDI in addition to standard care benefited greatly from a reduction in symptoms when compared to standard care. The improvement in symptoms could result in a quicker return to regular life [129]. Despite a wide range of advantages, there might appear potential side effects in LABA/LAMA therapy, such as cardiovascular events, including increased risk of myocardial infarction and stroke, as well as respiratory issues like dry mouth, cough, and paradoxical bronchospasm. Moreover, less common but serious adverse effects include acute angle glaucoma, urinary retention, and tachycardia, necessitating careful patient monitoring during treatment [130].

### 3.6. Monoclonal Antibodies

Monoclonal antibodies (mAbs) are biological drugs that exert their effects by specifically binding to target antigens with high affinity, thereby modulating the immune response. Their primary mechanism involves the neutralization of circulating antigens or blocking receptor–ligand interactions on immune or infected cells, which can alter signal transduction pathways essential for immune activation or viral propagation [131]. Additionally, mAbs can engage the immune system through their Fc regions, triggering antibody-dependent cellular cytotoxicity (ADCC) or complement-dependent cytotoxicity (CDC), which enhances the clearance of infected or aberrant cells. In the context of viral infections, mAbs may contribute to antiviral defense by preventing viral entry into host cells or by promoting the elimination of virus-infected cells via Fc-mediated immune mechanisms [132]. mAbs mimetic interact with receptors or ligands to modulate immune system function; mAb against COVID-19, such as casirivimab and imdevimab, have been used to treat severe SARS-CoV-2 virus infection. They neutralize the virus, which reduces the amount of virus in the body and reduces inflammation. According to Li et al. [133], mAbs targeting glycoproteins—hemagglutinin and neuraminidase, which play a prominent role in the process of influenza virus infection and release, can effectively prevent the spread of the virus. Drugs, such as tocilizumab and baricitinib, target specific cytokines or immune cells involved in the inflammatory response. Tocilizumab, an IL-6 receptor antagonist, has been shown to improve clinical outcomes in COVID-19 patients with severe inflammation [134]. Baricitinib, a Janus kinase (JAK) inhibitor, has also shown promise in reducing inflammation in COVID-19 patients. However, the use of immunomodulatory drugs in COVID-19 patients requires careful monitoring for potential side effects, such as infections and liver toxicity [135].

Omalizumab is a mAb that binds to the Fc fragment of IgE and is derived from humanized IgG1-κ. In RV infections, it decreased MMP concentration in bronchoalveolar lavage fluid and decreased reticular basement membrane thickness in adult subjects with severe asthma [136, 137]. Preincubating the cells with omalizumab inhibited both proliferation and matrix deposition [138]. Additionally, there is some evidence that this mAb can lessen fibronectin deposits in asthmatic airways and the thickness of the basement membrane [139].

Tezepelumab is a human IgG2*λ* mAb that binds to TSLP, a cytokine derived from epithelial cells that is linked to the pathophysiology of various asthma phenotypes, with a high affinity. When compared to placebo, tezepelumab administration was linked to less hyperresponsiveness to mannitol inhalation [140]. Tezelumab significantly increased FEV1 and decreased asthma exacerbations at week 52 according to the Phase III NAVIGATOR study [140].

In the second phase of CASCADE trial, a hypothesis was established that by blocking TSLP with tezepelumab, airway inflammation, hyperresponsiveness, and possibly airway remodeling would all be decreased [141]. However, the authors observed no effect on airway remodeling outcomes, though airway hyperresponsiveness was reduced with tezepelumab when compared to placebo.

It is important to emphasize that asthma biologics though discussed here for antiviral/anti-inflammatory properties are specifically not recommended for asthma flares or treatment of viral illness but rather for asthma control. That is why they should be distinguished from the immunomodulatory therapies discussed just previously, which have been used in acute viral illness (SARS-CoV-2). Among the many negative consequences that mAbs can cause are cytopenias, hepatotoxicity, infusion responses, dermatologic toxicities, and immune-related side effects such as progressive multifocal leukoencephalopathy and cytokine release syndrome. Depending on the precise target and mAb's mode of action, these adverse effects can change and are frequently dose-dependent [142].

## 4. Potential of Anti-Inflammatory Drugs in Preventing Airway Remodeling

Airway remodeling involves structural modifications to the walls of the airways and is a hallmark of long-term inflammatory conditions like asthma and COPD [5]. Anti-inflammatory drugs play a crucial role in managing these conditions. These medications can help stop or lessen the series of events that lead to airway remodeling by lowering inflammation. Controlling inflammation is essential to preventing additional harm because it is a major factor in the structural alterations seen in airway remodeling [143].

Inflammatory processes often trigger fibrotic responses, leading to increased collagen deposition and tissue remodeling in the airways. By interfering with these fibrotic pathways, anti-inflammatory medications can lessen the severity of airway remodeling and stop excessive collagen deposition. On the other hand, fibrosis may also be caused by many sets of factors [144]. Selecting the appropriate target to prevent fibrosis is therefore a challenging task that heavily depends on the particular type and stage of the disease as well as inflammation present in the tissue—fibrosis and tissue regeneration are both influenced by inflammation. However, inflammation is not a necessary condition for tissue regeneration or a guarantee that fibrosis will develop [145].

Several viruses, including RSV, RV, and influenza virus, are known to exacerbate airway inflammation and promote remodeling processes in the respiratory tract [17, 18, 146]. Antiviral drugs targeting these pathogens primarily act by directly inhibiting viral replication, thereby limiting the acute phase of infection and reducing virus-induced cytopathic effects [146]. This antiviral action, although not inherently anti-inflammatory, can indirectly mitigate airway inflammation and remodeling by preventing the prolonged immune activation typically driven by persistent viral presence. In contrast to anti-inflammatory agents, which exert direct immunomodulatory effects, the impact of antivirals on airway remodeling is largely secondary, mediated through their capacity to reduce the viral burden and its associated immune consequences [147]. Therefore, while antiviral therapy may not directly suppress inflammation, its timely administration can play a critical role in modulating downstream inflammatory cascades and tissue remodeling, especially when used early in the disease course or in combination with agents that target inflammation more specifically. This distinction underscores the importance of understanding both the primary and secondary mechanisms by which antiviral interventions may influence the long-term outcomes of chronic airway diseases [16].

Laboratory and clinical studies suggest that corticosteroids exert both direct anti-inflammatory effects and indirect effects on airway remodeling. Their main mechanism of action involves suppression of pro-inflammatory cytokine production, inhibition of inflammatory cell recruitment, and regulation of inflammatory gene expression through interaction with transcription factors such as NF-*κ*B and AP-1, leading to a reduction in airway inflammation [148]. In addition, corticosteroids may affect structural changes in the airways through indirect mechanisms. Long-term anti-inflammatory effects may lead to reduced smooth muscle cell proliferation, reduced collagen deposition, inhibition of fibroblast activity, and upregulation of apoptosis processes, which may ultimately reduce excessive mucosal thickness and improve airflow [148, 149]. In clinical trials such as the START study, Pauwels et al. [150] have demonstrated that early intervention with ICSs significantly reduces the rate of decline in lung function and attenuates airway remodeling over time. Similarly, the CAMP trial (The Childhood Asthma Management Program Research Group, 2000) showed that long-term use of ICSs in children reduced markers of inflammation, although structural changes were less conclusively reversed [151]. A 2024 cohort study analyzed the long-term safety of oral corticosteroids (OCS) in patients with COPD. The study found that prolonged use of OCS was associated with an increased risk of adverse outcomes, including osteoporosis, type 2 diabetes, cardiovascular/cerebrovascular diseases, and all-cause mortality. Notably, the risk of these adverse outcomes increased with higher cumulative OCS doses. For example, the risk of cardiovascular/cerebrovascular diseases was 34% higher in patients with cumulative OCS doses between 1.0 and 2.5 g compared to those with doses under 0.5 g [152]. A randomized controlled trial by Baraket et al. [153] highlighted that prolonged GC therapy can lead to permanent damage affecting various organs and systems, even after discontinuation. Notable complications include cataracts, skin atrophy, striae, acne, and obesity. These findings underscore the importance of limiting GC prescriptions to the lowest effective dose and duration to mitigate potential long-term risks; however, according to the authors, 200 μg/day of fluticasone propionate was as effective as 1000 μg/day in improving asthma control, airway inflammation, lung function, and AHR in adults. Despite the benefits, it is noteworthy; however, that standard doses of ICSs are ineffective in 5%–10% of patients with asthma who are insensitive or resistant to these drugs. In such cases, rather than simply increasing the dose of corticosteroids, it is necessary to reverse the mechanisms responsible for the reduction in maximal response to treatment before any therapeutic benefit becomes apparent [154].

Some studies have suggested that NSAIDs, particularly aspirin and certain selective COX-2 inhibitors, may exhibit both direct anti-inflammatory and indirect antiremodeling effects in asthma. Their primary mechanism involves the inhibition of COX enzymes, leading to a reduction in the synthesis of PGs, which are key mediators of inflammation [155]. This direct suppression of inflammatory pathways can alleviate acute symptoms and reduce immune cell activation. Indirectly, by modulating the chronic inflammatory environment and interfering with profibrotic signaling, NSAIDs may help limit structural changes in the airway wall, including subepithelial fibrosis and smooth muscle proliferation. However, the role of NSAIDs in airway remodeling remains controversial, particularly in individuals with aspirin-exacerbated respiratory disease (AERD), where their use can provoke severe bronchoconstriction [156]. Furthermore, the known systemic side effects of NSAIDs, such as cardiovascular and gastrointestinal risks, must be carefully weighed against their potential therapeutic benefits in this context.

While LABAs and LAMAs are not typically classified as antiremodeling agents, their pharmacological effects may still contribute to limiting structural changes in the airways [119]. Primarily, these drugs exert a direct bronchodilatory effect by relaxing airway smooth muscle—LABAs through *β*2-receptor activation and LAMAs through inhibition of muscarinic M3 receptors—thereby improving airflow and alleviating bronchoconstriction. This direct effect can lead to a reduction in mechanical stress on the airway wall, which is a recognized contributor to airway remodeling in chronic respiratory diseases [157]. Additionally, both drug classes have been reported to exhibit modest anti-inflammatory properties, though these effects are indirect and less potent compared to corticosteroids. Through the combination of reduced mechanical strain and secondary immunomodulatory actions, LABAs and LAMAs may help attenuate remodeling processes over time, particularly when used as part of combination therapy with ICS, which directly target airway inflammation and fibrotic signaling pathways [158, 159]. This distinction between their primary symptom-relieving action and secondary impact on disease progression is essential for understanding their role in long-term airway management.

Since IFNs are central to the innate immune response against viral infections, their therapeutic administration may influence airway remodeling primarily through indirect mechanisms [160]. By limiting viral replication and viral-induced inflammation, IFNs help reduce the frequency and severity of exacerbations in respiratory diseases, which in turn may prevent the chronic inflammatory milieu that promotes tissue remodeling [158]. Although IFNs can also modulate immune cell activation, cytokine production, and potentially interfere with fibrotic signaling pathways, these effects are generally considered secondary to their primary antiviral function. Unlike corticosteroids, which directly suppress inflammation and immune activation, IFNs exert their anti-inflammatory impact more indirectly—by controlling upstream triggers such as viral pathogens and restoring immune balance [161]. Consequently, while there is emerging evidence supporting their potential to attenuate airway remodeling, their clinical application remains limited and is typically reserved for severe or treatment-resistant cases rather than as a routine component of asthma or COPD management [162].

mAbs used in asthma therapy exert their primary effects through targeted immunomodulation by binding specific cytokines or immune receptors involved in the pathogenesis of type 2 inflammation. By directly neutralizing mediators such as IL-5, IL-4, IL-13, or IgE, these agents reduce eosinophilic infiltration, Th2 cell activation, and allergic inflammatory responses [163]. This direct anti-inflammatory action contributes to improved asthma control, fewer exacerbations, and enhanced lung function. The impact on airway remodeling, however, is largely indirect and secondary to inflammation control; by dampening chronic immune activation, mAbs may slow or attenuate processes such as subepithelial fibrosis, smooth muscle hypertrophy, or mucus gland hyperplasia. Additionally, some evidence suggests that mAbs may influence tissue-resident immune cells and modulate local cytokine microenvironments, potentially affecting the persistence of proremodeling stimuli. However, these effects are context-dependent and not uniform across all patient subgroups or antibody classes [164, 165]. Inhibiting IL-4 and IL-13 signaling, two important cytokines in the Th2 immune response, is another therapeutic target. IgE levels and eosinophil counts are also decreased by dupilumab, an antagonist of the same IL-4 receptor (IL-4R*α*), which also dramatically decreases the expression of IL-13-induced genes including POSTN (periostin). In addition to improving asthma management, lowering the activity of these cytokines also helps to reduce inflammation. According to some research, dupilumab may have a direct effect on smooth muscle cells in the airways, lowering their hyperreactivity, which is a crucial component of airway remodeling [166, 167].

Importantly, researchers do not directly reverse established structural alterations in the airways but may create a disease-modifying environment that prevents further architectural deterioration. Their use is typically reserved for patients with severe, treatment-refractory asthma, where sustained immune regulation can offer both symptomatic relief and long-term preservation of airway integrity, though ongoing research is exploring whether earlier intervention might yield more robust remodeling-related benefits.

## 5. The Advantages of Drug Combination Therapies

In clinical practice, the management of viral respiratory infections, particularly in asthmatic patients, often necessitates a multifaceted approach. The interplay between viral pathogens and the host immune system can exacerbate asthma symptoms and lead to airway remodeling. Therefore, combining antiviral agents with anti-inflammatory treatments is a strategic approach to mitigate these effects. Antiviral medications, such as neuraminidase inhibitors (e.g., oseltamivir), are commonly used to treat influenza infections. These agents inhibit the release of viral particles from infected cells, thereby limiting the spread of the virus within the respiratory tract. In patients with asthma, timely administration of antivirals can reduce the severity and duration of exacerbations triggered by viral infections [168].

Adding LAMAs, such as tiotropium, to ICS therapy has been shown to reduce the risk of exacerbations and improve lung function in patients with uncontrolled asthma. A systematic review and meta-analysis indicated that the combination therapy was associated with a 47% reduction in exacerbation risk compared to ICS alone. However, no significant difference in exacerbation risk was observed when compared to increasing the ICS dose or adding a LABA to ICS therapy. Furthermore, the addition of LAMAs did not significantly improve asthma control or quality of life scores compared to ICS/LABA therapy alone [169]. On the other hand, the concurrent use of NSAIDs with asthma medications has been associated with an increased risk of asthma exacerbations. A nationwide cohort study in Taiwan found that children with asthma who used NSAIDs had a 41% higher risk of hospitalization due to asthma exacerbations compared to those who did not use NSAIDs [170]. Similarly, a self-controlled case series study in Japan reported a significantly higher risk of acute asthma attacks during NSAID prescription periods, with nonselective NSAIDs posing a greater risk than COX-2 selective NSAIDs [171]

The combination of ICS and LABA is a standard treatment for asthma. A systematic review and meta-analysis of 20 randomized clinical trials involving 11,894 patients found that triple therapy (ICS, LABA, and LAMA) was significantly associated with fewer severe asthma exacerbations and modest improvements in asthma control compared to dual therapy (ICS plus LABA) [172]. Adding a LAMA to ICS therapy has shown benefits in asthma management. A comprehensive observational study indicated that the addition of LAMA to ICS therapy improved lung function and reduced asthma symptoms in patients with uncontrolled asthma [173]. Furthermore, a network meta-analysis of phase III studies reported that triple therapy with ICS, LABA, and LAMA reduced severe exacerbations and improved lung function compared to dual therapies [172]. The study by Rich et al. [174] indicated that corticosteroids might impair innate antiviral immune responses, potentially increasing the risk of exacerbations and severity of disease in asthma patients. This suppression of IFNs by corticosteroids could render patients more susceptible to viral infections, suggesting that type I and III IFN therapies might be beneficial in certain settings. Furthermore, research has shown that cytokine-induced steroid resistance in airway smooth muscle cells can occur through the upregulation of interferon regulatory factor-1 (IRF-1), which impairs steroid function. This mechanism highlights the complexity of combining corticosteroids with IFNs, as the presence of certain cytokines may reduce the efficacy of corticosteroids [175].

Personalized treatment strategies in respiratory diseases are becoming increasingly important, as they allow for tailored therapeutic approaches based on individual patient characteristics. In the context of respiratory tract remodeling, personalized anti-inflammatory treatments, such as corticosteroids or biologics, can be more effective by targeting specific molecular pathways and patient profiles. The use of biologics like mAbs (e.g., anti-IL-5 or anti-IL-4Rα) can provide significant benefits in patients with eosinophilic asthma, addressing the underlying inflammatory mechanisms and reducing airway remodeling. Moreover, genetic and biomarker profiling can help identify patients who are more likely to respond to certain anti-inflammatory therapies, improving clinical outcomes. Incorporating personalized treatment into clinical practice has the potential to reduce adverse effects, optimize drug efficacy, and prevent disease progression [4, 176].

## 6. Conclusions

Immune cells and signaling molecules interact intricately during inflammatory reactions in the airways. Anti-inflammatory medications have the ability to regulate these immune reactions, thereby impeding the recruitment and activation of inflammatory cells that are involved in the remodeling of airways [177]. Anti-inflammatory medications are helpful in maintaining airway function because they reduce inflammation and stop structural alterations in the airways. A personalized medicine approach to treating patients with severe asthma has been made possible by the introduction of several biological immunotherapies (e.g., anti-TSLP, anti-IgE). Anti-inflammatory medications have a great deal of promise for preventing airway remodeling, but it is important to understand that each patient will react differently and that a number of variables, including the severity of the disease, its underlying causes, and the patient's adherence to treatment, can affect how well a treatment works. Nevertheless, more investigation is required to clarify the best approaches for addressing airway remodeling and enhancing long-term results in individuals suffering from persistent inflammatory airway disorders.

## Data Availability

Data sharing is not applicable to this article as no new data were created or analyzed in this study.
